# Supplementary material for: Tuberculosis in individuals who recovered from COVID-19: A systematic review of case reports
Source: PLoS One. 2022 Nov 28;17(11):e0277807. doi: 10.1371/journal.pone.0277807 (PMC9704624; doi:10.1371/journal.pone.0277807)
Supplement: S2 Table — (DOCX) [file pone.0277807.s002.docx]

**Search engines**

**PubMed**

| Search number | Query | Sort By | Filters | Search Details | Results | Time |
| --- | --- | --- | --- | --- | --- | --- |
| 4 | (("Tuberculosis"[Mesh] OR "Latent Tuberculosis"[Mesh] OR "Extensively Drug-Resistant Tuberculosis"[Mesh] OR "Tuberculosis, Central Nervous System"[Mesh] OR "Tuberculosis, Multidrug-Resistant"[Mesh] OR "Tuberculosis, Urogenital"[Mesh] OR "Tuberculosis, Splenic"[Mesh] OR "Tuberculosis, Spinal"[Mesh] OR "Tuberculosis, Renal"[Mesh] OR "Tuberculosis, Pulmonary"[Mesh] OR "Tuberculosis, Pleural"[Mesh] OR "Tuberculosis, Osteoarticular"[Mesh] OR "Tuberculosis, Oral"[Mesh] OR "Tuberculosis, Ocular"[Mesh] OR "Tuberculosis, Miliary"[Mesh] OR "Tuberculosis, Meningeal"[Mesh] OR "Tuberculosis, Male Genital"[Mesh] OR "Tuberculosis, Lymph Node"[Mesh] OR "Tuberculosis, Laryngeal"[Mesh] OR "Tuberculosis, Hepatic"[Mesh] OR "Tuberculosis, Gastrointestinal"[Mesh] OR "Tuberculosis, Female Genital"[Mesh] OR "Tuberculosis, Endocrine"[Mesh] OR "Tuberculosis, Cardiovascular"[Mesh] OR "Tuberculosis, Bovine"[Mesh] OR "Tuberculosis, Cutaneous"[Mesh] OR "Mycobacterium tuberculosis"[Mesh]) AND ("COVID-19"[Mesh] OR "SARS-CoV-2"[Mesh])) AND ("Case Reports" [Publication Type]) | | | ("Tuberculosis"[MeSH Terms] OR "Latent Tuberculosis"[MeSH Terms] OR "Extensively Drug-Resistant Tuberculosis"[MeSH Terms] OR "tuberculosis, central nervous system"[MeSH Terms] OR "tuberculosis, multidrug resistant"[MeSH Terms] OR "tuberculosis, urogenital"[MeSH Terms] OR "tuberculosis, splenic"[MeSH Terms] OR "tuberculosis, spinal"[MeSH Terms] OR "tuberculosis, renal"[MeSH Terms] OR "tuberculosis, pulmonary"[MeSH Terms] OR "tuberculosis, pleural"[MeSH Terms] OR "tuberculosis, osteoarticular"[MeSH Terms] OR "tuberculosis, oral"[MeSH Terms] OR "tuberculosis, ocular"[MeSH Terms] OR "tuberculosis, miliary"[MeSH Terms] OR "tuberculosis, meningeal"[MeSH Terms] OR "tuberculosis, male genital"[MeSH Terms] OR "tuberculosis, lymph node"[MeSH Terms] OR "tuberculosis, laryngeal"[MeSH Terms] OR "tuberculosis, hepatic"[MeSH Terms] OR "tuberculosis, gastrointestinal"[MeSH Terms] OR "tuberculosis, female genital"[MeSH Terms] OR "tuberculosis, endocrine"[MeSH Terms] OR "tuberculosis, cardiovascular"[MeSH Terms] OR "tuberculosis, bovine"[MeSH Terms] OR "tuberculosis, cutaneous"[MeSH Terms] OR "Mycobacterium tuberculosis"[MeSH Terms]) AND ("COVID-19"[MeSH Terms] OR "SARS-CoV-2"[MeSH Terms]) AND "Case Reports"[Publication Type] | 55 | 4:12:46 |
| 3 | "Case Reports" [Publication Type] | Most Recent |  | "Case Reports"[Publication Type] | 2,294,141 | 4:11:38 |
| 2 | "COVID-19"[Mesh] OR "SARS-CoV-2"[Mesh] | Most Recent |  | "COVID-19"[MeSH Terms] OR "SARS-CoV-2"[MeSH Terms] | 189,333 | 4:11:12 |
| 1 | "Tuberculosis"[Mesh] OR "Latent Tuberculosis"[Mesh] OR "Extensively Drug-Resistant Tuberculosis"[Mesh] OR "Tuberculosis, Central Nervous System"[Mesh] OR "Tuberculosis, Multidrug-Resistant"[Mesh] OR "Tuberculosis, Urogenital"[Mesh] OR "Tuberculosis, Splenic"[Mesh] OR "Tuberculosis, Spinal"[Mesh] OR "Tuberculosis, Renal"[Mesh] OR "Tuberculosis, Pulmonary"[Mesh] OR "Tuberculosis, Pleural"[Mesh] OR "Tuberculosis, Osteoarticular"[Mesh] OR "Tuberculosis, Oral"[Mesh] OR "Tuberculosis, Ocular"[Mesh] OR "Tuberculosis, Miliary"[Mesh] OR "Tuberculosis, Meningeal"[Mesh] OR "Tuberculosis, Male Genital"[Mesh] OR "Tuberculosis, Lymph Node"[Mesh] OR "Tuberculosis, Laryngeal"[Mesh] OR "Tuberculosis, Hepatic"[Mesh] OR "Tuberculosis, Gastrointestinal"[Mesh] OR "Tuberculosis, Female Genital"[Mesh] OR "Tuberculosis, Endocrine"[Mesh] OR "Tuberculosis, Cardiovascular"[Mesh] OR "Tuberculosis, Bovine"[Mesh] OR "Tuberculosis, Cutaneous"[Mesh] OR "Mycobacterium tuberculosis"[Mesh] | Most Recent |  | "Tuberculosis"[MeSH Terms] OR "Latent Tuberculosis"[MeSH Terms] OR "Extensively Drug-Resistant Tuberculosis"[MeSH Terms] OR "tuberculosis, central nervous system"[MeSH Terms] OR "tuberculosis, multidrug resistant"[MeSH Terms] OR "tuberculosis, urogenital"[MeSH Terms] OR "tuberculosis, splenic"[MeSH Terms] OR "tuberculosis, spinal"[MeSH Terms] OR "tuberculosis, renal"[MeSH Terms] OR "tuberculosis, pulmonary"[MeSH Terms] OR "tuberculosis, pleural"[MeSH Terms] OR "tuberculosis, osteoarticular"[MeSH Terms] OR "tuberculosis, oral"[MeSH Terms] OR "tuberculosis, ocular"[MeSH Terms] OR "tuberculosis, miliary"[MeSH Terms] OR "tuberculosis, meningeal"[MeSH Terms] OR "tuberculosis, male genital"[MeSH Terms] OR "tuberculosis, lymph node"[MeSH Terms] OR "tuberculosis, laryngeal"[MeSH Terms] OR "tuberculosis, hepatic"[MeSH Terms] OR "tuberculosis, gastrointestinal"[MeSH Terms] OR "tuberculosis, female genital"[MeSH Terms] OR "tuberculosis, endocrine"[MeSH Terms] OR "tuberculosis, cardiovascular"[MeSH Terms] OR "tuberculosis, bovine"[MeSH Terms] OR "tuberculosis, cutaneous"[MeSH Terms] OR "Mycobacterium tuberculosis"[MeSH Terms] | 227,910 | 4:10:24 |

**CINAHL**

**
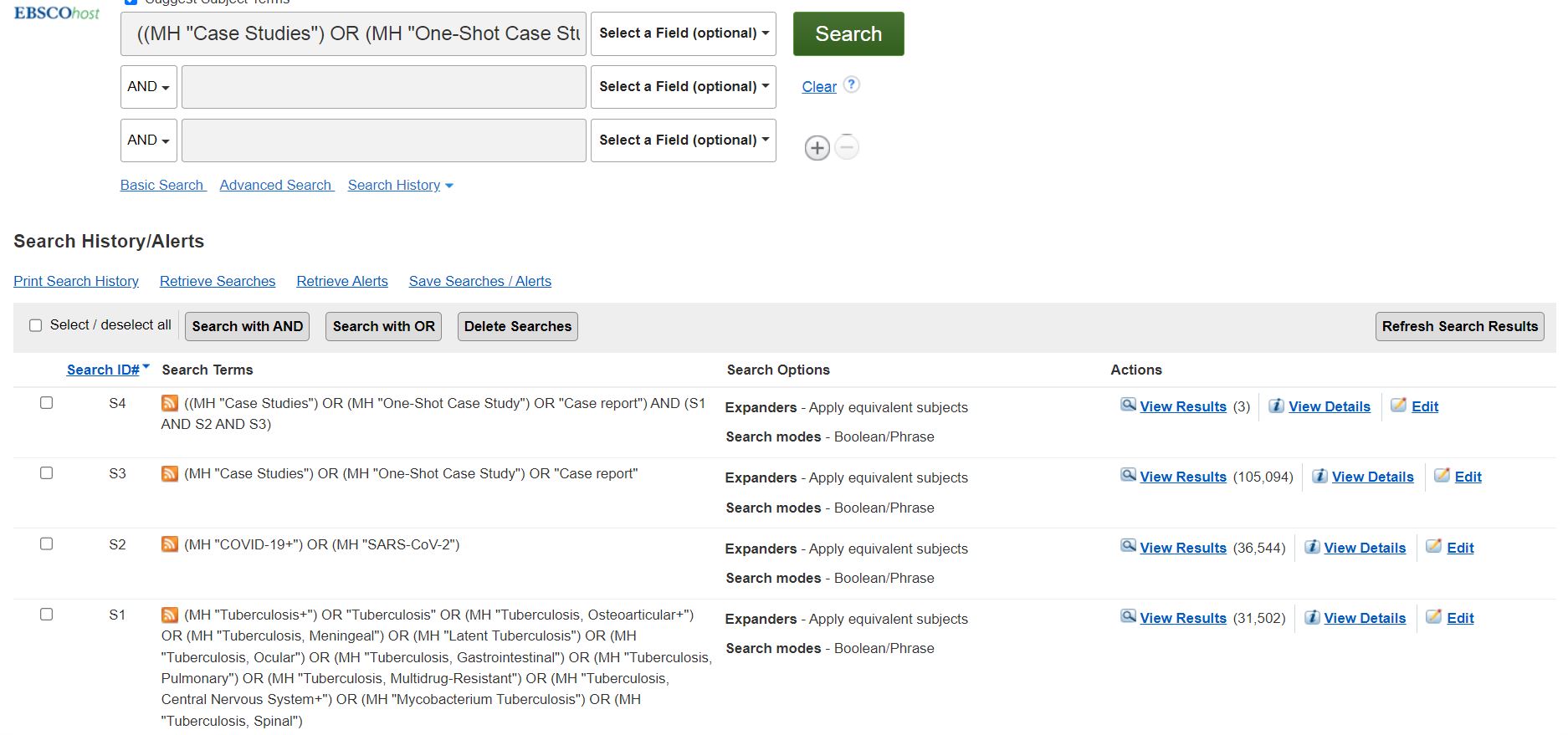
**

**
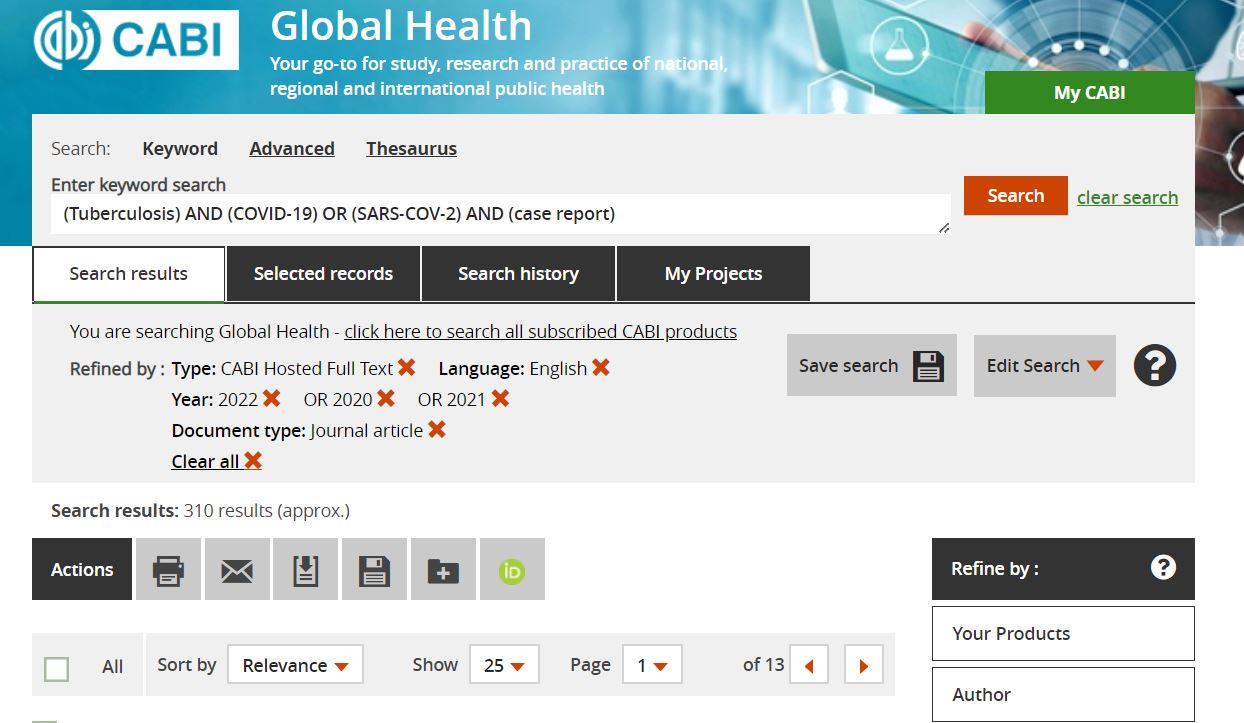
**

**
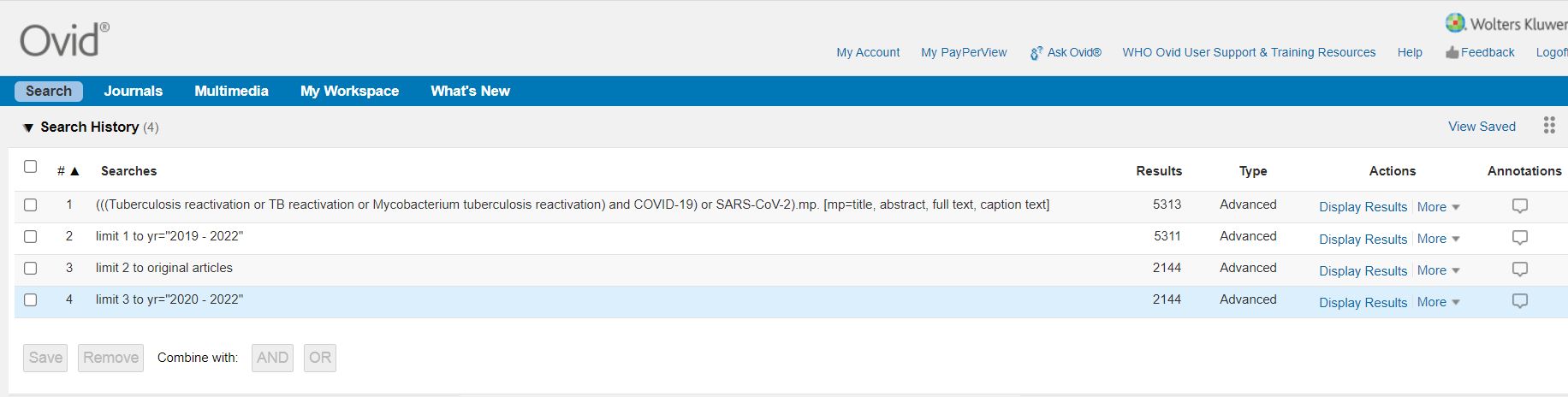
**

**
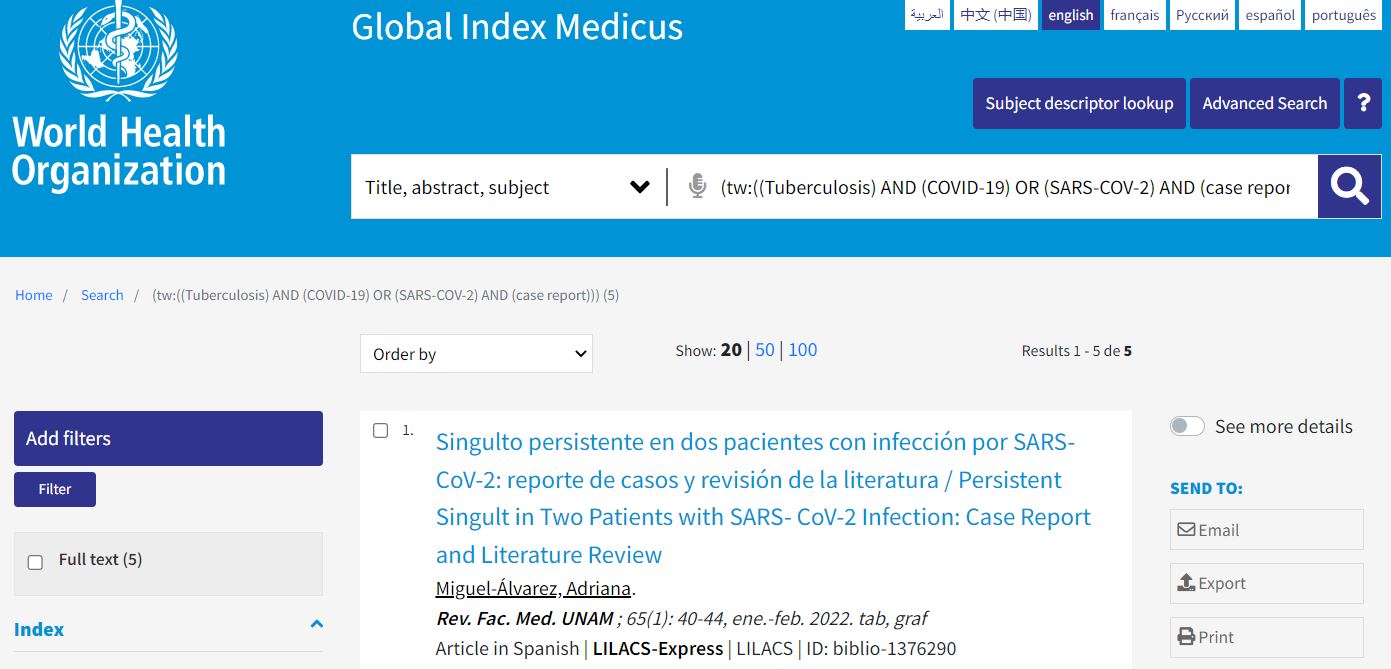
**
