# Supplementary material for: Tuberculosis in individuals who recovered from COVID-19: A systematic review of case reports
Source: PLoS One. 2022 Nov 28;17(11):e0277807. doi: 10.1371/journal.pone.0277807 (PMC9704624; doi:10.1371/journal.pone.0277807)
Supplement: S3 Table — (DOCX) [file pone.0277807.s003.docx]

**Quality assessment for the included studies in meta-analysis**

Critical appraisal for case reports

| Author, Year | Q1 | | | | Q2 | | | | Q3 | | | | | Q4 | | | | Q5 | | | | Q6 | | | | Q7 | | | | Q8 | | | | Quality score |
| --- | --- | --- | --- | --- | --- | --- | --- | --- | --- | --- | --- | --- | --- | --- | --- | --- | --- | --- | --- | --- | --- | --- | --- | --- | --- | --- | --- | --- | --- | --- | --- | --- | --- | --- |
|  | Y | N | U | NA | Y | N | U | NA | Y | N | U | NA | Y | | N | U | NA | Y | N | U | NA | Y | N | U | NA | Y | N | U | NA | Y | N | U | NA |  |
| Khayat et al., 2021 | √ |  |  |  |  | √ |  |  | √ |  |  |  | √ | |  |  |  | √ |  |  |  |  | √ |  |  |  |  |  | √ | √ |  |  |  | 62.5% |
| Ntshalintshali et al., 2021 | √ |  |  |  |  | √ |  |  | √ |  |  |  | √ | |  |  |  | √ |  |  |  | √ |  |  |  |  |  |  | √ | √ |  |  |  | 75% |
| Unal et al., 2021 | √ |  |  |  | √ |  |  |  | √ |  |  |  | √ | |  |  |  | √ |  |  |  | √ |  |  |  |  |  |  | √ | √ |  |  |  | 87.5% |
| Lee et al., 2021 | √ |  |  |  | √ |  |  |  | √ |  |  |  | √ | |  |  |  | √ |  |  |  | √ |  |  |  |  |  |  | √ | √ |  |  |  | 87.5% |
| Elmoqaddem et al., 2020 | √ |  |  |  | √ |  |  |  | √ |  |  |  | √ | |  |  |  |  | √ |  |  |  | √ |  |  |  |  |  | √ | √ |  |  |  | 62.5% |
| Pozdnyakov et al., 2021 | √ |  |  |  |  | √ |  |  | √ |  |  |  | √ | |  |  |  | √ |  |  |  | √ |  |  |  |  |  |  | √ | √ |  |  |  | 75% |
| Grang and Lee, 2020 | √ |  |  |  | √ |  |  |  | √ |  |  |  | √ | |  |  |  | √ |  |  |  | √ |  |  |  |  |  |  | √ | √ |  |  |  | 87.5% |
| Aguillón‑Durán et al., 2021, | √ |  |  |  | √ |  |  |  | √ |  |  |  | √ | |  |  |  |  | √ |  |  |  | √ |  |  |  |  |  | √ | √ |  |  |  | 62.5% |
| Dahanayake et al., 2020 | √ |  |  |  | √ |  |  |  | √ |  |  |  | √ | |  |  |  | √ |  |  |  | √ |  |  |  |  |  |  | √ | √ |  |  |  | 87.5% |
| Zahid et al., 2021 | √ |  |  |  | √ |  |  |  | √ |  |  |  | √ | |  |  |  | √ |  |  |  | √ |  |  |  |  |  |  | √ | √ |  |  |  | 87.5% |
| Podder and Chowdhury, 2020 | √ |  |  |  | √ |  |  |  | √ |  |  |  | √ | |  |  |  | √ |  |  |  | √ |  |  |  |  |  |  | √ | √ |  |  |  | 87.5% |
| Asif et al., 2021 | √ |  |  |  |  | √ |  |  | √ |  |  |  | √ | |  |  |  | √ |  |  |  | √ |  |  |  |  |  |  | √ | √ |  |  |  | 75% |
| Win et al., 2021 | √ |  |  |  |  | √ |  |  | √ |  |  |  | √ | |  |  |  | √ |  |  |  | √ |  |  |  |  |  |  | √ | √ |  |  |  | 75% |
| Noh and Dronavalli, 2021 | √ |  |  |  | √ |  |  |  | √ |  |  |  | √ | |  |  |  | √ |  |  |  |  | √ |  |  |  |  |  | √ | √ |  |  |  | 75% |
| Elziny et al., 2021 | √ |  |  |  | √ |  |  |  | √ |  |  |  | √ | |  |  |  | √ |  |  |  | √ |  |  |  |  |  |  | √ | √ |  |  |  | 87.5% |
| Burda etal., 2021 | √ |  |  |  |  | √ |  |  | √ |  |  |  | √ | |  |  |  | √ |  |  |  |  | √ |  |  |  |  |  | √ | √ |  |  |  | 62.5% |
| Cutler et al., 2020 | √ |  |  |  | √ |  |  |  | √ |  |  |  | √ | |  |  |  | √ |  |  |  | √ |  |  |  |  |  |  | √ | √ |  |  |  | 87.55 |
| Younest al., 2022 | √ |  |  |  | √ |  |  |  | √ |  |  |  | √ | |  |  |  |  | √ |  |  |  | √ |  |  |  |  |  | √ | √ |  |  |  | 62.5% |
| Guliani et al., 2021 | √ |  |  |  |  | √ |  |  | √ |  |  |  | √ | |  |  |  | √ |  |  |  | √ |  |  |  |  |  |  | √ | √ |  |  |  | 75% |
| Rahimi et al., 2022 | √ |  |  |  |  | √ |  |  | √ |  |  |  | √ | |  |  |  | √ |  |  |  | √ |  |  |  |  |  |  | √ | √ |  |  |  | 75% |
| Leonso et al., 2022 | √ |  |  |  | √ |  |  |  | √ |  |  |  | √ | |  |  |  | √ |  |  |  | √ |  |  |  |  |  |  | √ | √ |  |  |  | 87.5% |

***Y=yes, N=no, U=unclear, NA=not applicable, <60%=low,60-80%=medium, >80%=high quality***
