# Supplementary material for: Tuberculosis in individuals who recovered from COVID-19: A systematic review of case reports
Source: PLoS One. 2022 Nov 28;17(11):e0277807. doi: 10.1371/journal.pone.0277807 (PMC9704624; doi:10.1371/journal.pone.0277807)
Supplement: Attachment — Submitted filename: Response to reviewers.docx [file pone.0277807.s005.docx]

PONE-D-22-18569R1

Tuberculosis in individuals who recovered from COVID-19: A Systematic Review of Case Reports

**Editor comments**

Journal Requirements:
Please review your reference list to ensure that it is complete and correct. If you have cited papers that have been retracted, please include the rationale for doing so in the manuscript text, or remove these references and replace them with relevant current references. Any changes to the reference list should be mentioned in the rebuttal letter that accompanies your revised manuscript. If you need to cite a retracted article, indicate the article’s retracted status in the References list and also include a citation and full reference for the retraction notice.
**Response:** **Thank you for the pertinent comment. All the included references are available in the web and in the revised manuscript we have included the link (DOI) for the studies**.

Additional Editor Comments:
All queries have been addressed. However, there are some minor corrections.
Line 28- 'resulted' may be replaced by 'resulting'

**Response: Thank you, it is replaced.**
Lines 28/29- delete 'due to'

**Response: Thank you, it is deleted.**
Line 30- delete 'it'

**Response: Thank you, it is deleted.**
Line 39, 239- delete 'were'

**Response: Thank you, it is deleted.**
Line 39- HIV need not be in italics

**Response: Thank you, it is corrected**
Line 41, 180- 'any' to be replaced by 'some'

**Response: Thank you, we made a replacement**
Line 64- insert 'it was' before 'observed'

**Response: Thank you, we made a change accordingly**
Line 77- 'recovery' instead of 'recovered'

**Response: Thank you, we changed it**
Line 176- 'is' to be replaced by 'was', 'so' to be inserted before 'that'

**Response: Thank you, revised per the suggestion.**
Line 179- It should be '16' patients instead of '17' as total number of patients is 33

**Response: Thank you, it was a clerical error. Now, it is corrected.**
Line 186- Instead of 'nine', antibody to SARS CoV2 was positive in '11' cases. As per your Table 2.

**Response: Thank you. Antibody to SARS CoV2 was positive in 11 cases. As displayed in table 2 among 8 patients in the same study (patient 3-patient 10), antibody was positive in 6 patients and for the remaining 2 cases it was not described. Thus including the remaining 3 cases it becomes nine.**

Line 245- It is not clear how being HIV seronegative indicates diminished immune status

**Response: Thank you, we have revised it. Our assumption was that the immune suppressed status of COVID-19 infected individuals might led to developing TB. This is a common phenomenon in HIV positive individuals.**
Line 249- 'as a' may be replaced by 'in'

**Response: Thank you, we made a replacement per the comment.**
